# Myeloid Focal Adhesion Kinase Promotes Macrophage Accumulation but Does Not Alter Tumor Progression in Hepatocellular Carcinoma

**DOI:** 10.1002/mc.70156

**Published:** 2026-07-26

**Authors:** Eugene Ham, Kyle Boedeker, Claudia Rose Keating, Ruisong Ye, Frankie Montalbano, Sophia Mankaruse, Xianzhong Ding, Jonathan P. Rennhack, Wei Qiu

**Affiliations:** 1 Department of Surgery, Loyola University Chicago Stritch School of Medicine, Maywood, Illinois, USA; 2 Department of Cancer Biology, Loyola University Chicago Stritch School of Medicine, Maywood, Illinois, USA; 3 Department of Pathology and Laboratory Medicine, Loyola University Chicago Stritch School of Medicine, Maywood, Illinois, USA

**Keywords:** LysM-Cre, MET/β-catenin, *PTK2*, single-cell transcriptomics, tumor microenvironment

## Abstract

Hepatocellular carcinoma (HCC) develops within an immunologically complex tumor microenvironment that is heavily shaped by infiltrating myeloid cells. Immune-based treatment strategies such as atezolizumab plus bevacizumab have shown promising therapeutic benefits, but patients do not experience durable responses. This shortfall motivates current efforts to elucidate signaling programs that sustain pro-tumor myeloid states. Focal adhesion kinase (FAK, encoded by *PTK2*) integrates adhesion, growth factor, proliferative, and inflammatory signaling, but the contribution of FAK in myeloid cells to HCC has not been tested. In this study, we used human HCC transcriptomic analyses and a myeloid-specific FAK knockout mouse model to investigate the role of myeloid-intrinsic FAK in HCC progression. Analysis of the PRHCCdb single-cell database showed *PTK2* expression was detectable in myeloid populations. Higher *PTK2* expression in myeloid-rich TCGA-LIHC tumors was associated with a significantly worse overall survival. Further analysis of the NCI-CLARITY single-cell data set localized the strongest FAK-associated transcriptional programs to a monocyte-derived macrophage state marked by *VCAN* and *COLEC12*. Specifically, this population demonstrated hypoxia-associated and inflammatory signatures. To test the functional role of FAK in myeloid cells, we generated myeloid-specific FAK knockout (LysMCre;FAK^f/f^) mice. Deletion of FAK did not alter baseline liver morphology, liver-to-body weight ratio, or proliferation. When challenged with the MET/β-catenin-driven HCC model via hydrodynamic tail vein injection, myeloid-specific FAK deletion did not significantly affect survival, gross tumor burden, or tumor proliferation. However, tumors from the LysMCre;FAK^f/f^ mice showed significantly reduced F4/80^+^ macrophage accumulation compared with FAK^f/f^ controls. These findings indicate that myeloid-specific FAK promotes macrophage accumulation, but its loss alone is insufficient to alter tumor burden, proliferation, or survival in this oncogene-driven HCC model.

## 1 ∣ Introduction

Hepatocellular carcinoma (HCC) is the predominant form of primary liver cancer. It is partly shaped by the immunological complexity of the tumor microenvironment (TME), where immune, stromal, endothelial, and extracellular matrix (ECM) components interact to promote tumor growth and suppress antitumor immunity [1–3]. Although atezolizumab plus bevacizumab and other immune-based regimens have improved outcomes for patients, many tumors resist or progress during therapy [4, 5]. These challenges motivate the search for new treatment strategies in HCC, which increasingly center on the immune cell populations that remodel the TME and promote disease progression.

Tumor-associated macrophages (TAMs) are among the most abundant immune cells in HCC and have been broadly implicated in disease progression from angiogenesis and immune suppression to therapeutic resistance [6–8]. Single-cell transcriptomic studies have further revealed that macrophages are not a uniform population but rather comprise multiple states with distinct inflammatory, hypoxic, and vascular transcriptional programs [9, 10]. Which signaling pathways regulate these states in HCC, and whether targeting them can meaningfully offer a prognostic benefit, remains to be determined.

Focal adhesion kinase (FAK), a nonreceptor tyrosine kinase encoded by *PTK2*, is one such candidate that may connect macrophage biology to tumor progression. FAK canonically integrates signals downstream of integrins and growth factor receptors to regulate adhesion, migration, survival, and cyto-skeletal remodeling [11–13]. FAK has also been reported to influence NF-κB activation via IKKα phosphorylation [14]. In the TME, FAK has been shown to regulate immune cell recruitment, response to checkpoint blockade therapy, angiogenesis, and ECM remodeling [15, 16]. In HCC, prior work from our group demonstrated that hepatocyte-specific FAK deletion suppresses MET/β-catenin (MET/CAT)-driven hepatocarcinogenesis and prolongs survival, supporting a tumor-promoting role for FAK in hepatocytes [17, 18]. Recent studies have also shown that FAK inhibition strategies can improve the efficacy of anti-PD-1 responses, primarily acting on angiogenesis, fibrosis, immunosuppressive cell infiltration, and CD8^+^ T cell infiltration [19–21]. Still, the majority of this work has focused on tumor-intrinsic FAK activity or systemic FAK inhibition. How FAK in myeloid cells contributes to HCC remains unexplored.

This distinction is relevant because macrophages depend on adhesion, chemotaxis, and inflammatory signaling to infiltrate tumors, and myeloid-specific FAK has been shown to regulate these processes [22]. At the same time, TAMs in HCC produce angiogenic factors including VEGF, PDGF, and matrix metallo-proteinases [6], particularly under hypoxic conditions where HIF-1α-dependent signaling drives pro-angiogenic gene expression [23]. These observations raise the possibility that FAK in myeloid cells contributes to HCC by shaping macrophage-driven inflammatory and hypoxia-associated angiogenic programs.

In this study, we first mapped *PTK2* expression across human HCC using single-cell and bulk transcriptomic data sets and identified a *PTK2*-enriched macrophage state associated with hypoxia and inflammatory transcriptional signatures. We then generated myeloid-specific FAK knockout (LysMCre;FAK^f/f^) mice and challenged them with MET/CAT-driven HCC using the Sleeping Beauty transposon system, a model that re-capitulates an aggressive and clinically relevant molecular subtype of HCC [24]. By pairing human HCC transcriptomic evidence with a myeloid-specific knockout model, this study investigates the functional relevance of myeloid-specific FAK (hereafter myeloid FAK) in HCC progression.

## 2 ∣ Materials and Methods

### 2.1 ∣ Mice

All animals used for experiments received humane care in accordance with the “Guide for the Care and Use of Laboratory Animals” (https://www.ncbi.nlm.nih.gov/books/NBK54050/). All procedures were reviewed and approved by Loyola University Chicago Institutional Animal Care and Use Committee. LysMCre;FAK^f/f^ mice were generated by crossing LysMCre mice (JAX, Cat# 018956) with FAK^f/f^ mice (MMRRC, Cat# 009967). The resulting LysMCre;FAK^f/+^ offspring were then crossed with FAK^f/f^ mice to generate LysMCre;FAK^f/f^ and FAK^f/f^ littermate controls. All mice were housed in micro-isolator cages under a 12-h light/dark cycle and were given access to water and chow ad libitum. Genotyping was performed using the primers listed in Table 1.

### 2.2 ∣ Hydrodynamic Tail Vein Injection

The MET/CAT-induced HCC model was performed as described previously [25], outlined in Figure 5A. In brief, age-matched mice were subjected to hydrodynamic tail vein injection (HTVi) for a total of 50 μg of plasmids consisting of 22.5 μg pSB-EF1α > hMET + 22.5 μg pSB-EF1α > ΔN90-β-catenin + 5 μg Sleeping Beauty transposase (HSB2). The plasmids were diluted in sterile 0.9% saline at a volume equivalent to 10% of their body weight (e.g., 2 mL for a 20 g mouse) and injected rapidly via tail vein injection within 5–7 s for FAK^f/f^ (*n* = 8) and LysMCre;FAK^f/f^ (*n* = 6) mice. For oncogene expression validation, mice were sacrificed 7 days after injection, and livers were collected for western blot analysis. For survival studies, mice were monitored and euthanized when they met humane endpoint criteria. Abdominal circumference measurements were obtained using a flexible measuring tape wrapped around the abdomen. Plasmid information is listed in Table 2.

### 2.3 ∣ Isolation of Bone Marrow Cells

Eight-week-old mice were euthanized (*n* = 3 per genotype), and bilateral femurs and tibias were collected under sterile conditions. After removal of the surrounding muscle and cartilage, bone marrow was flushed using a syringe with a 27-gauge, ½-inch needle filled with ice-cold PBS. The bone marrow suspension was centrifuged, washed with PBS, and treated with ACK lysis buffer to lyse red blood cells for 7 min at room temperature. Lysis was stopped by adding ice-cold PBS at 10 times the volume of the lysis buffer. Following centrifugation, cells were resuspended in RPMI-1640 medium and plated for adherence. The following day, cells were supplemented with 20 ng/mL mouse recombinant granulocyte-macrophage colony-stimulating factor (GM-CSF). Fresh GM-CSF-containing medium was replenished daily for 5 days. Cells were then harvested and subjected to western blot analysis to confirm FAK deletion. Reagents used are listed in Table 2.

### 2.4 ∣ Western Blotting

Western blot analysis was performed as previously described [25]. Information about the primary and secondary antibodies can be found in Table 2.

### 2.5 ∣ Hematoxylin and Eosin Staining

Liver tissues were fixed in zinc-buffered formalin for 24 h and then transferred to 70% ethanol for storage. Samples were submitted to VitroVivo Biotech (Rockville, MD) for embedding. Paraffin-embedded tissues were sectioned at 5 μm and dried on a slide rack overnight. The following day, sections were stained with hematoxylin and eosin, mounted, and imaged using an Olympus BX41 microscope with an Olympus DP21 camera at 100× and 200× magnifications. The H&E-stained sections were reviewed by a board-certified pathologist blinded to genotype.

### 2.6 ∣ Immunohistochemistry Staining

Paraffin-embedded sections were prepared as described above and baked at 55°C for 2 h. Sections were deparaffinized, rehydrated, and subjected to citrate-based antigen retrieval. After cooling to room temperature, endogenous peroxidase activity was quenched with 3% hydrogen peroxide in TBS for 5 min in the dark. Sections were washed in TBS and blocked with 20% goat serum in TBS for 1 h at room temperature. After blocking, sections were rinsed in 0.05% TBS-T and incubated overnight at 4°C with primary antibodies diluted 1:100 (Ki67, F4/80) or 1:200 (CD31) in 5% goat serum/TBS (antibody details outlined in Table 2). The following day, slides were equilibrated to room temperature for 30 min, washed in 0.05% TBS-T, and incubated with biotin-conjugated secondary antibodies diluted 1:100 in 5% goat serum/TBS for 1 h at room temperature. Sections were washed and incubated with ABC-HRP reagent for 30 min in the dark. Signal was developed using DAB substrate until dark-brown staining was observed (approximately 2–4 min). Slides were rinsed in ddH2O, counterstained with hematoxylin, clarified, blued, dehydrated, and mounted.

Stained sections were imaged on an Olympus BX41 microscope with an Olympus DP21 camera. Quantification of signal was done with 10 random nonoverlapping fields per sample captured at 200× magnification unless otherwise stated. Images were analyzed in ImageJ using the Color Deconvolution 2 plugin [26, 27]. Ki67 index was calculated as the percentage of Ki67-positive nuclei among total nuclei. F4/80 and CD31 staining was quantified as the percentage of F4/80- and CD31-positive area relative to total tissue area. Values were averaged per sample. Reagents used are listed in Table 2.

### 2.7 ∣ scRNA-Seq Analysis

**FIGURE 1 ∣:**
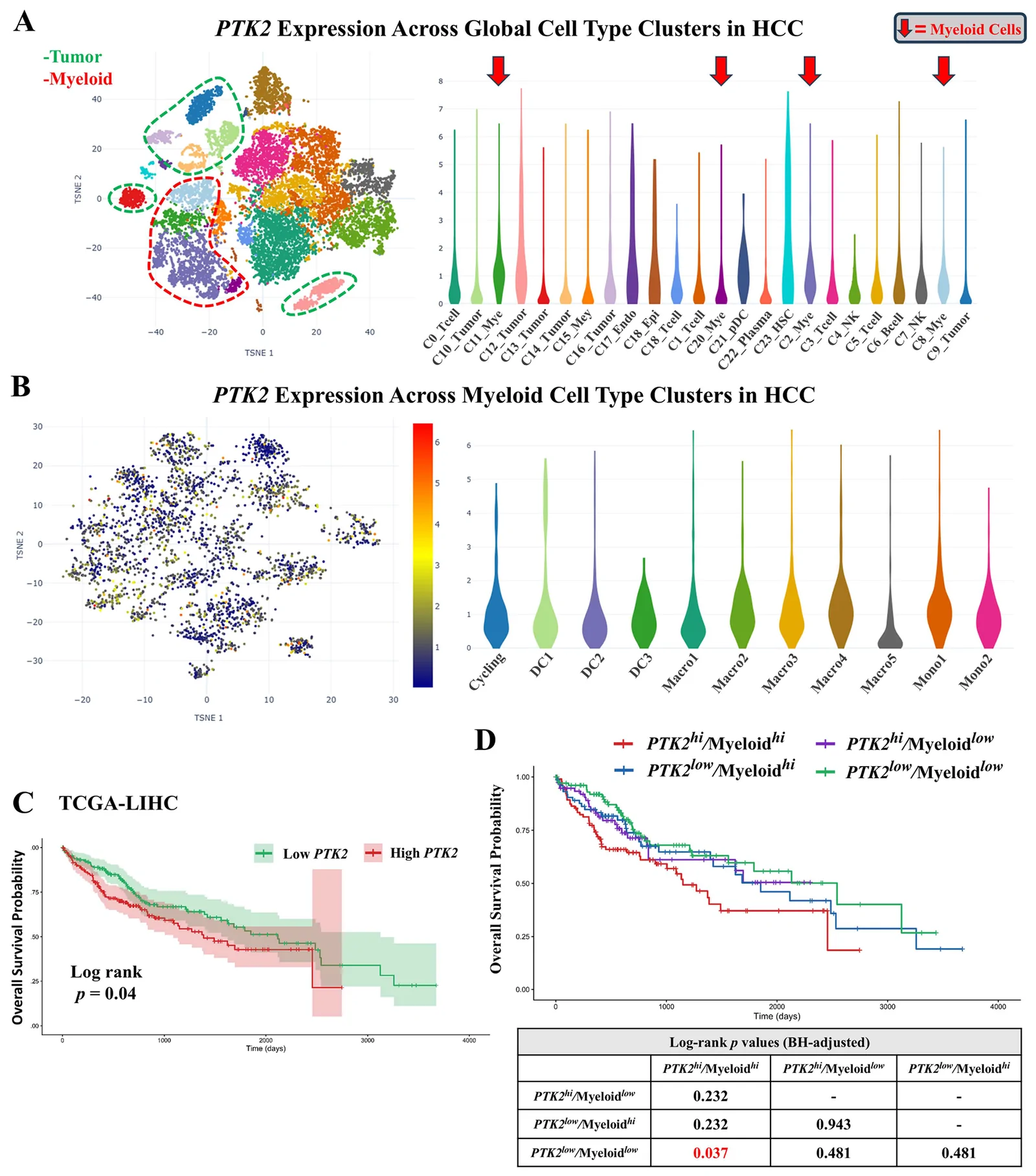
*PTK2* is enriched in myeloid cells and associates with worse survival in myeloid-rich HCC. (A) UMAP of global cell populations from the PRHCCdb online database. Tumor and myeloid compartments are indicated with green and red dotted circles, respectively. Violin plots depict *PTK2* expression across the cell types. (B) UMAP of myeloid cell subclusters with *PTK2* expression overlaid. Violin plots depict *PTK2* expression across myeloid subtypes. (C) Kaplan–Meier analysis of TCGA-LIHC patients stratified by median *PTK2* expression (*n* = 183 per group); log-rank *p* = 0.04. (D) Kaplan–Meier analysis of TCGA-LIHC patients stratified by median *PTK2* expression and TIMER2.0-derived myeloid infiltration score. xCell-derived myeloid scores were used as a sensitivity analysis for myeloid infiltration stratification. Pairwise log-rank *p* values (BH-adjusted) are reported in the table. [Color figure can be viewed at wileyonlinelibrary.com]

The *PTK2^+^* cell type annotations in Figure 1A,B were derived from a publicly available PRHCCdb resource (https://db.cngb.org/PRHCCdb/). This resource provides pre-annotated single-cell RNA-sequencing (scRNA-seq) data from primary and early-relapse HCC, which we used as supplied and did not perform de novo analysis [28]. scRNA-seq data from the NCI-CLARITY HCC data set (GSE151530) [10] were manually analyzed using Seurat in R [29]. Filtered count matrices were loaded via Read10X. Ambient RNA contamination was assessed by evaluating hepatocyte-associated transcript expression in myeloid clusters and addressed through the removal of contaminated clusters during myeloid reclustering. Doublets were identified and removed using scDblFinder with sample-aware classification, where metadata were available [30]. Cells were then filtered to retain those with 300–6000 detected genes, fewer than 30,000 RNA counts, and less than 15% mitochondrial reads. SCTransform normalization with mitochondrial percentage regressed out was followed by principal component analysis, nearest-neighbor graph construction, clustering, and UMAP embedding [31]. Global cell-type annotations were assigned using canonical markers and supported by SingleR with Human Primary Cell Atlas and Blueprint/ENCODE references [32]. The NCI-CLARITY data set contains 46 biopsies (32 HCC and 14 intrahepatic cholangiocarcinoma, iCCA) from 37 patients. HCC and iCCA samples were retained in the global analysis to maximize statistical power for cell-type identification. A sensitivity analysis restricted to HCC-derived cells (1919 of 2341 myeloid cells) confirmed concordant results for the *VCAN*^+^/*COLEC12*^+^ macrophage state.

For myeloid analyses, *C1QC^+^* Kupffer-like macrophages and inflammatory monocytes were subsetted and reclustered. Subclusters were annotated through canonical marker expression, SingleR annotation, DotPlot and FeaturePlot validation, and differential marker analysis (genes used outlined in Table 3). Contaminant, unresolved, and hepatocyte-enriched clusters were removed. Residual *CHGA^+^* cells were also excluded in all myeloid subclusters, yielding 6 validated myeloid populations for downstream analyses.

To avoid pseudo-replication bias, differential expression between myeloid populations was performed at the donor level using pseudo-bulk aggregation [33–35]. Raw RNA counts per donor and cluster were aggregated (*VCAN*^+^/*COLEC12*^+^ vs. other myeloid) and pseudo-bulk samples < 10 cells were excluded, which yielded 21 donor-level aggregates. Differential expression was tested via DESeq2 (FDR < 0.05, ∣log_2_FC∣ > 0.5) using an unpaired design [36]. To check for concordance between donor and cell-level differential expression, Wilcoxon rank-sum tests were used, with genes meeting adjusted *p* < 0.05 and ∣log_2_FC∣ > 0.5 considered significant. For pathway enrichment, gene set enrichment analysis (GSEA) was performed using fgsea [37] on the pseudo-bulk DESeq2-ranked gene lists against the Hallmark, KEGG, and Reactome collections from MSigDB [38, 39]. FAK pathway module scores were calculated using a FAK-associated gene set outlined in Table 3. The FAK module score correlation analysis (Figure 3D) ranks genes by association with a continuous score rather than a two-group testing and is reported as a complementary analysis. Ligand–receptor interactions were inferred by CellChat using secreted signaling, ECM–receptor, and cell–cell contact categories [40]. Cell groups < 10 cells were excluded.

### 2.8 ∣ TCGA-LIHC Analysis

TCGA-LIHC RNA-seq and clinical data were obtained using TCGAbiolinks in R [41]. STAR-count expression data were downloaded from the Genomic Data Commons, retaining only primary tumor samples. Gene-level counts were converted to transcripts-per-million (TPM) using union-exon lengths from EnsDb.Hsapiens.v86. Duplicate gene symbols were collapsed by summing counts, and *PTK2* expression was extracted from the resulting matrix. Overall survival was calculated from days to death or last follow-up, and survival status was derived from vital status.

Tumor immune infiltration was estimated from TPM data using immunedeconv [42]. Myeloid infiltration scores were derived from TIMER estimates of macrophages, neutrophils, and myeloid dendritic cells [43]. xCell-derived myeloid scores, dichotomized at the median, were used as a sensitivity analysis for the myeloid infiltration stratification, with concordance between methods assessed by Spearman’s correlation [44]. Patients were stratified by the median of the TIMER-derived myeloid infiltration score (high vs. low) and by the median *PTK2* expression. ESTIMATE-derived tumor purity scores were also incorporated as a continuous covariate to control for tumor composition bias [45]. Where multiple aliquots existed per patient, values were collapsed to the patient level using the median.

Kaplan–Meier analyses were performed using the survival and survminer packages, with patients stratified by median *PTK2* expression and myeloid infiltration score for visualization. Survival differences were assessed using log-rank test with pairwise comparisons adjusted via Benjamini–Hochberg (BH). Cox proportional hazards models were fitted as univariable and multivariable models, the latter adjusted for age, sex, pathologic stage, and tumor purity, along with interaction models testing the joint effect of *PTK2* and myeloid infiltration on survival. Proportional hazards assumptions were evaluated using Schoenfeld residuals. Where violations were detected, tumor purity was categorized into tertiles in sensitivity analyses.

### 2.9 ∣ Statistical Analysis

Statistical analyses were conducted using RStudio or GraphPad Prism software. Data are presented as mean ± SEM unless otherwise stated. Comparison between two groups used unpaired two-tailed *t*-tests with Welch’s correction. In the single-cell analysis, one-way multiple group comparisons were assessed using the Kruskal–Wallis test followed by Dunn’s post hoc test where indicated. For factorial groups in the IHC analyses, a two-way ANOVA with Holm–Šídák-corrected post hoc tests was used. TCGA-LIHC survival analysis used the Cox proportional hazards modeling and Kaplan–Meier curves with the log-rank test. Pairwise survival comparisons were adjusted using the BH method where indicated. For scRNA-seq analysis, differential expression was determined using pseudo-bulk aggregation and DESeq2, with a cell-level Wilcoxon rank-sum test used as a concordance check. Genes with adjusted *p* < 0.05 and ∣log_2_FC∣ > 0.5 were considered significant. GSEA was performed using ranked gene lists. Pathway significance was evaluated using FDR-adjusted values. A *p* < 0.05 was considered statistically significant.

## 3 ∣ Results

### 3.1 ∣ Human HCC Data Sets Associate Myeloid *PTK2* With Poor Prognosis

To determine the cellular context of *PTK2* expression in human HCC, we first used the PRHCCdb online resource (https://db.cngb.org/PRHCCdb/) and observed *PTK2* expression across multiple cellular compartments, including tumor and myeloid cells (Figure 1A). Within the myeloid compartment, *PTK2* expression was distributed across different myeloid subclusters (Figure 1B). To evaluate the clinical relevance of *PTK2* expression, we performed Kaplan–Meier survival analysis using the TCGA-LIHC data set. Patients with high *PTK2* expression had significantly worse overall survival than those with low *PTK2* expression (Figure 1C). We further stratified TCGA-LIHC tumors by *PTK2* expression and myeloid infiltration scores derived from TIMER2.0, with xCell used as a sensitivity analysis [43, 44]. Dual stratification by median *PTK2* expression and myeloid infiltration score revealed that patients with both high *PTK2* expression and high myeloid infiltration had significantly worse overall survival compared to those with low *PTK2* expression and low myeloid infiltration (BH-adjusted *p* = 0.037; Figure 1D). To quantify these associations, we fit Cox proportional-hazards models treating *PTK2* expression and myeloid infiltration as continuous variables. Both higher *PTK2* expression and higher myeloid infiltration were independently associated with worse overall survival (univariable HR = 1.29 per SD, 95% CI 1.09–1.53, *p* = 0.003; and HR = 1.20 per SD, 1.05–1.38, *p* = 0.009, respectively). Both remained significant after adjusting for age, sex, stage, and tumor purity (HR = 1.23, 1.02–1.49, *p* = 0.030; and HR = 1.23, 1.04–1.46, *p* = 0.018, respectively). Their interaction was not significant (*p* = 0.31), indicating that the two are each prognostic but do not synergize statistically. Myeloid scores from TIMER2.0 and xCell were moderately correlated (Spearman’s *ρ* = 0.51, *p* < 0.001), and the *PTK2*-survival association was preserved using xCell-derived scores (HR = 1.30, *p* = 0.005), confirming the result was not specific to a single deconvolution method. These findings suggest that *PTK2* expression and myeloid infiltration co-occur in a poor-prognosis subset of HCC. We therefore sought to identify the specific myeloid populations in which FAK-associated transcriptional programs are most prominent.

### 3.2 ∣ scRNA-Seq Analysis Identifies a *PTK2*-Enriched Macrophage State

**FIGURE 2 ∣:**
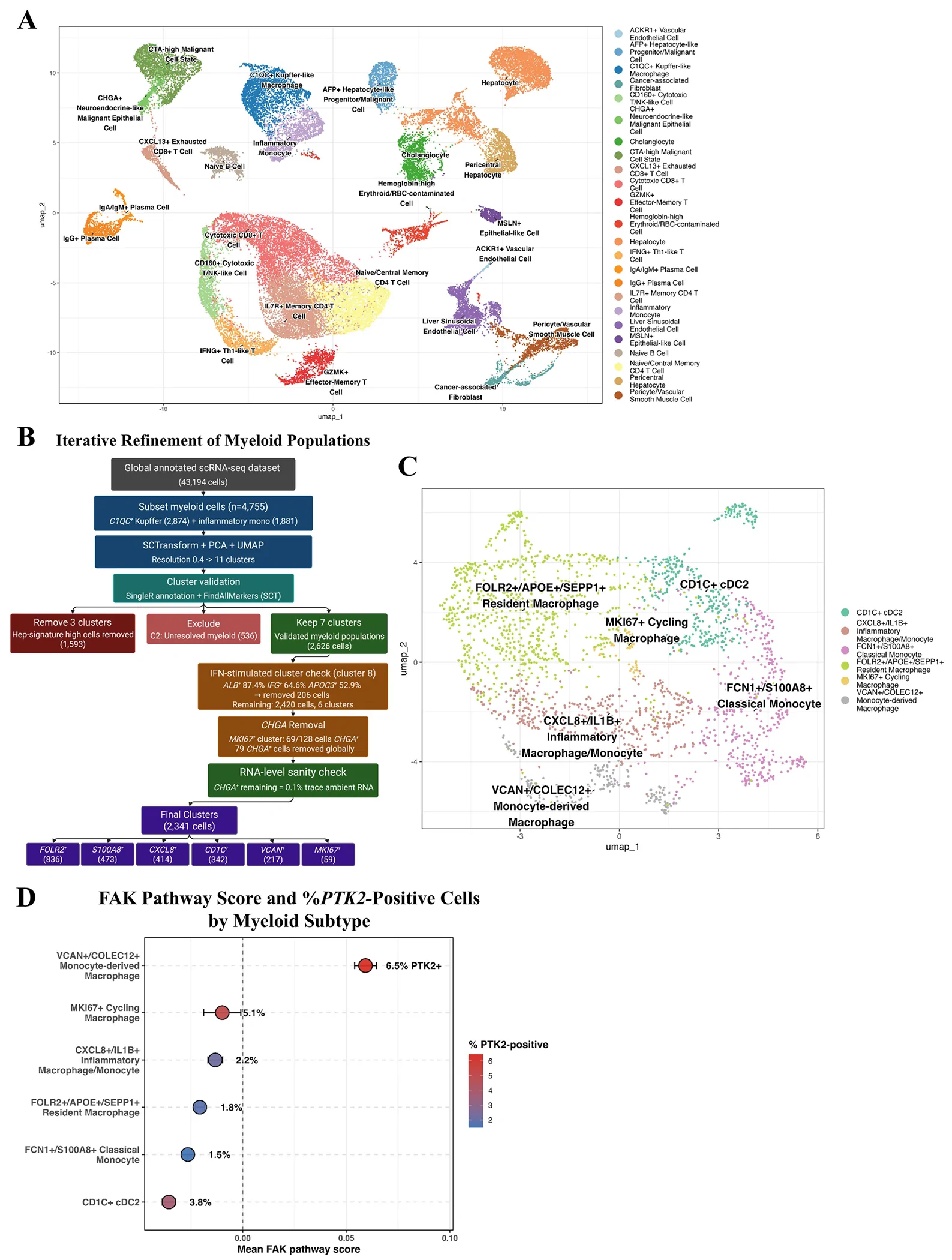
scRNA-seq analysis identifies a *PTK2*-enriched monocyte-derived macrophage population in HCC. (A) UMAP of annotated cell populations from the NCI-CLARITY data set after quality control, doublet removal, and cell-type annotation supported by SingleR classifications. (B) Schematic of iterative myeloid reclustering and quality control pipeline. After contamination removal through the outlined steps, six clusters remained and were utilized for downstream analyses. (C) UMAP of refined myeloid subclusters. (D) Mean FAK pathway module score across myeloid subtypes. Kruskal–Wallis test (*p* = 7.53 × 10^−47^); Dunn post hoc significant for *VCAN*^+^/*COLEC12*^+^ macrophages against other subtypes. Dot color indicates percentage of *PTK2*- positive cells per cluster (color scale, right). Data presented as mean ± SEM. [Color figure can be viewed at wileyonlinelibrary.com]

To localize the myeloid *PTK2* signal more precisely, we analyzed the NCI-CLARITY single-cell data set [10], which includes 46 biopsies (32 HCC and 14 iCCA) from 37 patients. After quality control, the global data set contained 43,194 annotated cells across major malignant, immune, stromal, and endothelial compartments (Figure 2A). We subsetted the myeloid cells and performed reclustering to refine the myeloid compartment. This process identified 11 clusters, some of which showed evidence of contamination or ambiguous identity. We applied a stepwise filtering strategy that removed these contaminants, unresolved, and hepatocyte-enriched clusters. Six validated myeloid subclusters were identified for downstream analysis (Figure 2B,C). These included the *FOLR2^+^/APOE^+^/SEPP1^+^* resident macrophages, *FCN1^+^/S100A8^+^* classical monocytes, *CXCL8^+^/IL1B^+^* inflammatory macrophage/monocytes, *CD1C^+^* cDC2, *MKI67^+^* cycling macrophages, *and VCAN^+^/COLEC12^+^* monocyte-derived macrophages (hereafter referred to as *VCAN*^+^ macrophages). The *VCAN^+^* macrophage cluster showed the highest mean FAK pathway module score and the highest fraction of *PTK2* expression among the myeloid subclusters (Figure 2D). The *VCAN*^+^ macrophage cluster was 99.5% HCC-derived, and an HCC-restricted sensitivity analysis preserved this cluster with the highest FAK pathway enrichment. These results identify *VCAN^+^* macrophages as the myeloid population most strongly associated with FAK pathway transcriptional enrichment in human HCC.

### 3.3 ∣ *PTK2*-Enriched Macrophages Display Hypoxia-Associated and Inflammatory Transcriptional Programs

**FIGURE 3 ∣:**
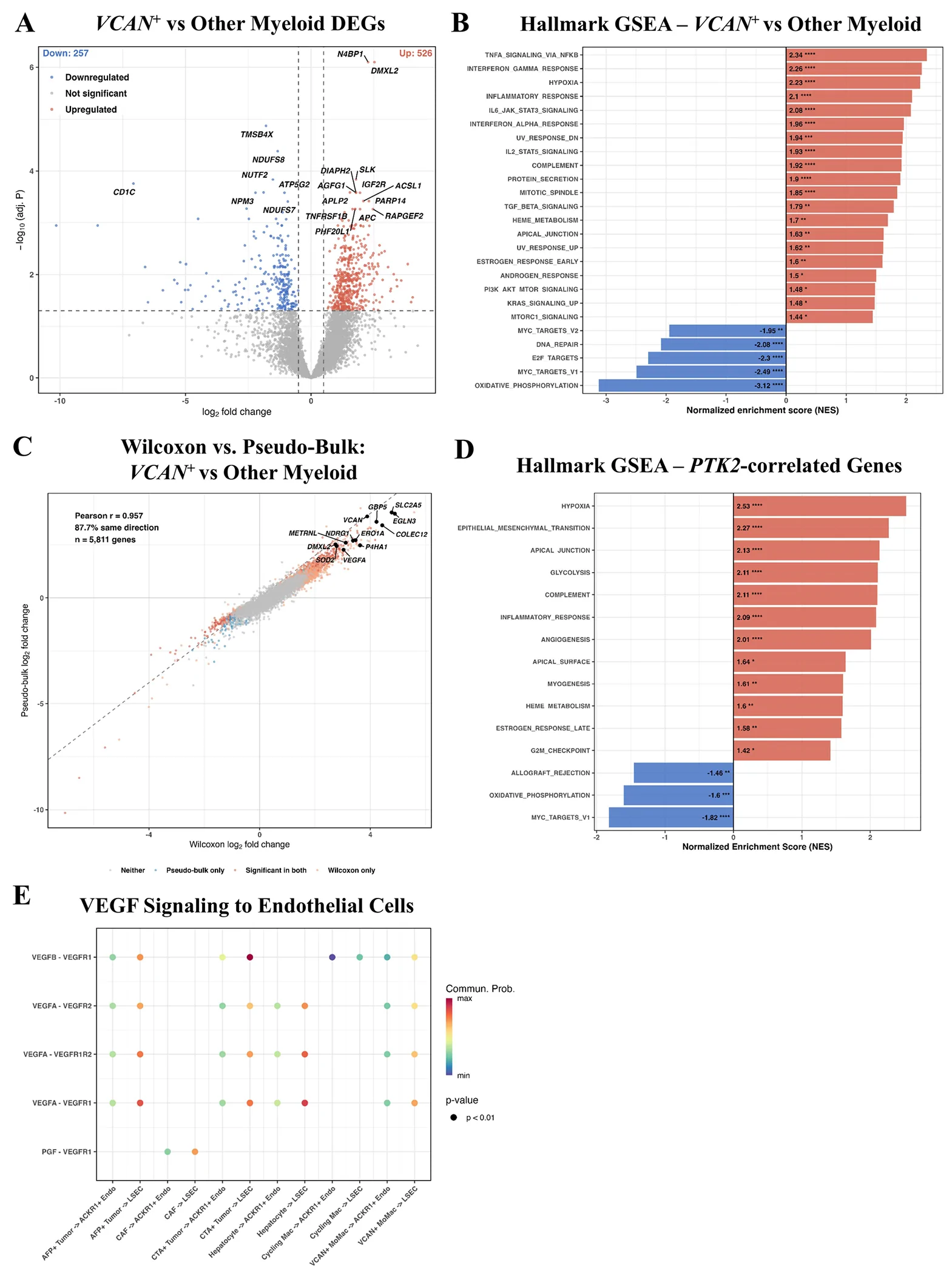
*PTK2*-associated myeloid programs converge on hypoxia, matrix remodeling, and angiogenic signaling. (A) Volcano plot showing differentially expressed genes in *VCAN*^+^/*COLEC12*^+^ macrophages compared with all other myeloid subclusters. Differential expression determined using DESeq2. Genes with FDR < 0.05 and ∣log_2_FC∣ > 0.5 were considered significant. Ribosomal genes were excluded from the labels. (B) Hallmark GSEA of the pseudo-bulk DESeq2 results, *VCAN*^+^/*COLEC12*^+^ macrophages against other myeloid subclusters. (C) Concordance of effect sizes between donor-level pseudo-bulk (DESeq2) and cell-level (Wilcoxon) differential expression. Pearson’s *r* = 0.957; 87.7% directional agreement across 5811 shared genes. (D) Hallmark GSEA of genes ranked by Spearman correlation with FAK pathway module scores across myeloid cells. Highlights pathways enriched among genes correlated with FAK pathway module score. (E) CellChat bubble plot of VEGF signaling from myeloid and tumor microenvironment sources to endothelial cells. Analyses were performed using ECM-receptor, secreted signaling, and cell–cell contact interaction categories. Communication probabilities computed using TriMean and cell groups filtered for at least 10 cells. Asterisks indicate statistical significance levels: *p < 0.05, **p < 0.01, ***p < 0.001, and ****p < 0.0001. [Color figure can be viewed at wileyonlinelibrary.com]

We next asked whether the *PTK2*- enriched macrophage state was associated with distinct transcriptional programs. Donor-level pseudo-bulk differential expression analysis comparing *VCAN^+^* macrophages with other myeloid populations (DESeq2; 21 samples with 2 *VCAN*^+^ and 19 others) identified genes associated with hypoxia, inflammatory signaling, metabolism, and tissue remodeling, including *SOD2, P4HA1, EGLN3, ERO1A, PDK1, TNFRSF1B, GBP5*, and *COLEC12* (Figure 3A). Hallmark GSEA showed enrichment of TNF-α/NF-κB signaling, interferon-γ response, hypoxia, inflammatory response, and IL6/JAK/STAT3 signaling. Oxidative phosphorylation and MYC target signatures were depleted (Figure 3B). To validate that this *VCAN^+^/COLEC12^+^* signature was not an artifact of the analytical method, we compared the pseudo-bulk analysis with the cell-level Wilcoxon analysis. Fold-change estimates were highly concordant between the two methods (Pearson’s *r* = 0.957), and cluster markers VCAN and COLEC12 remained significant. This corroborated that the *VCAN^+^/COLEC12^+^* signature is a robust biological signal (Figure 3C).

To determine whether these signatures extended beyond a single macrophage cluster and reflected broader FAK-associated myeloid programs, we ranked genes by their correlation with the FAK pathway module score across all myeloid cells. Genes positively correlated with FAK-associated transcriptional signatures were enriched for hypoxia, epithelial–mesenchymal transition, glycolysis, inflammatory response, angiogenesis, and myogenesis-associated signatures (Figure 3D). Given that hypoxia was enriched by GSEA in *VCAN^+^* macrophages, angiogenesis was enriched among *PTK2*-correlated myeloid programs, and hypoxic TAMs have been reported as a source of pro-angiogenic signaling [23], we used CellChat to infer ligand-receptor interactions between myeloid and endothelial populations. VEGF signaling from tumor and myeloid sources to endothelial targets was predicted, including *VEGFA*–VEGFR1 and *VEGFA*–VEGFR1/2 interactions from *VCAN^+^* macrophages to liver sinusoidal and *ACKR1^+^* vascular endothelial cells (Figure 3E). For these interactions, communication probabilities were 7.39 × 10^−6^ and 4.80 × 10^−6^ for *VEGFA*–VEGFR1 and *VEGFA*–VEGFR1/2 signaling to liver sinusoidal endothelial cells, and 1.94 × 10^−7^ and 1.74 × 10^−7^ for the corresponding interactions to *ACKR1^+^* vascular endothelial cells, with permutation *p* values reported as under 0.001 for all four interactions. Together, these findings describe a *PTK2*-enriched macrophage state characterized by hypoxia-associated, matrix remodeling, and inflammatory transcriptional signatures in HCC. This prompted us to investigate whether myeloid FAK functionally contributes to HCC progression.

### 3.4 ∣ Myeloid FAK Deletion Does Not Alter Baseline Liver Homeostasis

**FIGURE 4 ∣:**
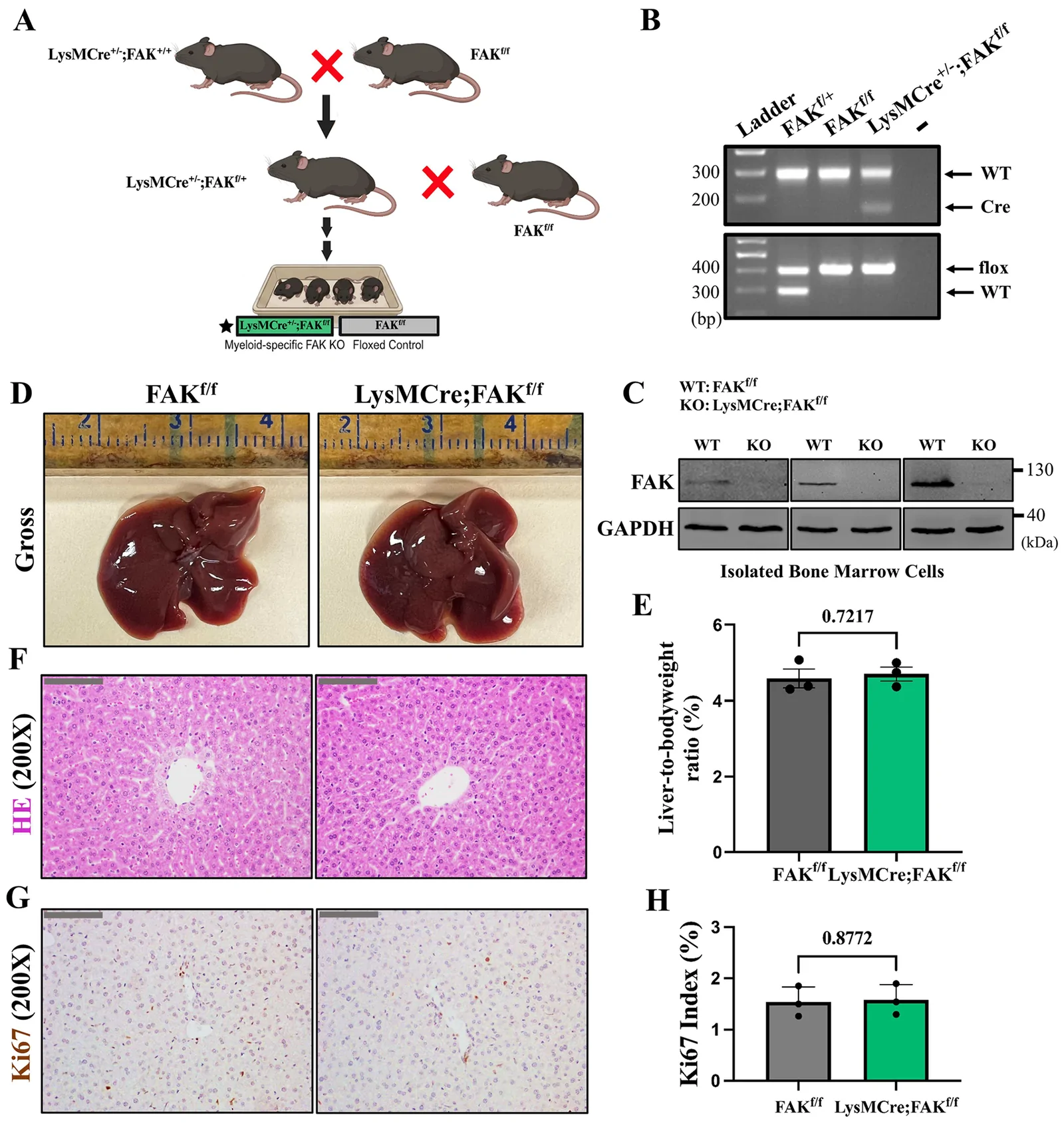
Myeloid FAK deletion does not alter baseline liver homeostasis. (A) Breeding strategy schematic for generating FAK^f/f^ and LysMCre;FAK^f/f^ mice. (B) Representative genotyping PCR from tail snips confirming indicated genotypes. (C) Western blot of FAK in isolated bone marrow cells pulsed with GM-CSF for 5 days from FAK^f/f^ and LysMCre;FAK^f/f^ mice (*n* = 3 per genotype). (D) Representative gross liver images. (E) Liver-to-body weight ratio (%), *n* = 3 per genotype. Unpaired two-tailed *t*-test used; *p* = 0.7217. (F) Representative H&E-stained liver sections. (G) Representative Ki67 IHC images of liver sections. (H) Quantification of Ki67 index (percentage of Ki67-positive nuclei among total nuclei per field), *n* = 3 per genotype. Unpaired two-tailed *t*-test used; *p* = 0.8772. IHC quantification was performed at 10 random nonoverlapping 200× fields per sample, analyzed via ImageJ. Scale bars = 100 μm. Data are presented as mean ± SEM, with each data point representing one individual sample. [Color figure can be viewed at wileyonlinelibrary.com]

To evaluate the contribution of myeloid FAK to HCC, we generated myeloid-specific FAK knockout (LysMCre;FAK^f/f^) mice using the breeding strategy outlined (Figure 4A). Genotyping of tail DNA confirmed the presence of the expected LysM-Cre transgene and floxed alleles (Figure 4B). To verify deletion of FAK in LysM-expressing cells, total bone marrow cells were cultured with GM-CSF. This approach promotes monocyte/macrophage differentiation and maturation of murine bone marrow cells, while also inducing FAK expression [46]. Western blot analysis confirmed markedly reduced FAK expression in bone marrow-derived cells from LysMCre;FAK^f/f^ mice compared with FAK^f/f^ control mice (Figure 4C). At baseline, deletion of FAK in myeloid cells did not alter liver morphology (Figure 4D) or the liver-to-body weight ratios between genotypes (*p* = 0.7217; Figure 4E). H&E staining further showed preserved hepatic architecture (Figure 4F). Ki67 staining and quantification showed no significant difference in baseline hepatocyte proliferation between genotypes (*p* = 0.8772; Figure 4G,H). These data demonstrate that myeloid FAK deletion does not alter baseline liver homeostasis.

### 3.5 ∣ Myeloid FAK Deletion Does Not Alter MET/β-Catenin-Driven HCC Development

**FIGURE 5 ∣:**
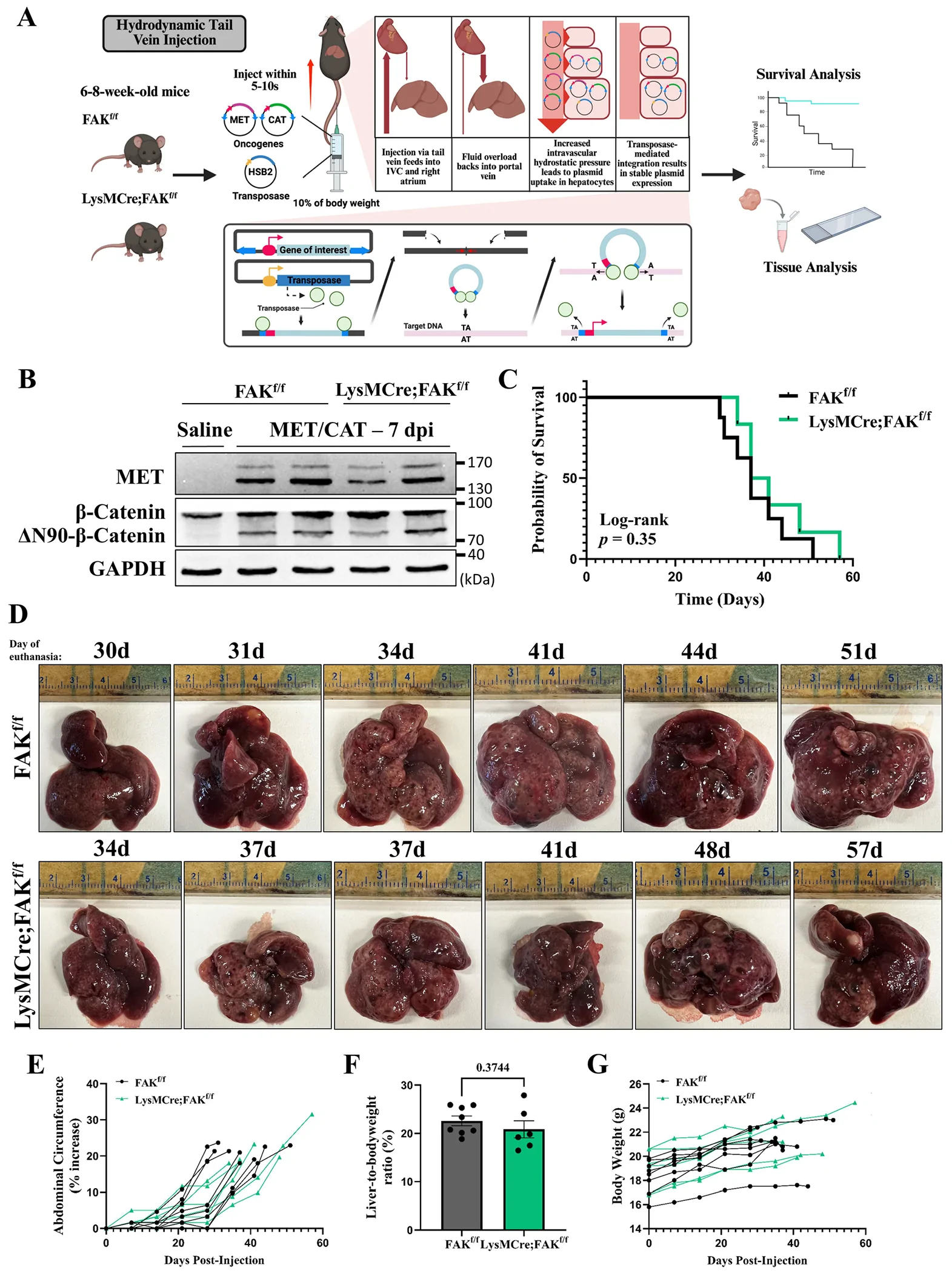
Myeloid FAK deletion does not alter MET/β-catenin-driven HCC development. (A) Schematic of hydrodynamic tail vein injection of MET and β-catenin (CAT) using the Sleeping Beauty transposon system. (B) Western blot confirming efficient gene delivery to the liver in both FAK^f/f^ and LysMCre;FAK^f/f^ mice (*n* = 2 per genotype) following HTVi. (C) Kaplan–Meier survival analysis of MET/CAT-injected mice; FAK^f/f^ (*n* = 8), LysMCre;FAK^f/f^ (*n* = 6); log-rank *p* = 0.35. (D) Representative gross liver images at indicated time points post-HTVi. (E) Descriptive percent increase in abdominal circumference for each mouse over time. (F) Liver-to-body weight ratio (%) at endpoints. Unpaired two-tailed *t*-test used; *p* = 0.3744. (G) Longitudinal body weight measurements for each mouse over time post-HTVi. Data are presented as mean ± SEM, with each data point representing one individual sample. [Color figure can be viewed at wileyonlinelibrary.com]

To investigate the role of myeloid FAK in hepatocarcinogenesis, we induced MET/CAT-driven HCC in LysMCre;FAK^f/f^ mice and littermate FAK^f/f^ controls using the Sleeping Beauty transposon system and HTVi (Figure 5A). Western blot analysis of injected livers from both genotypes at 7 days postinjection confirmed expression of MET and β-catenin/ΔN90-β-catenin, indicating comparable delivery of oncogenes (Figure 5B). Despite the TCGA-LIHC association between *PTK2*, myeloid infiltration, and survival, myeloid FAK deletion did not significantly alter overall survival (log-rank *p* = 0.35; Figure 5C). Both genotypes developed large, multifocal liver tumors with comparable gross appearance (Figure 5D). Longitudinal abdominal circumference measurements showed a largely similar en-largement pattern between both genotypes (Figure 5E). Quantification of endpoint liver-to-body weight ratio was also not significantly different (*p* = 0.3744; Figure 5F). The body weights of mice from both genotypes showed a similar increase over time, ruling out cachexia as a confounding factor of the liver-to-body weight ratio (Figure 5G). Collectively, these findings reveal that myeloid FAK deletion alone is not sufficient to modify overall tumor development in MET/CAT-induced HCC.

### 3.6 ∣ Myeloid FAK Deletion Reduces Macrophage Accumulation Without Altering Tumor Proliferation or Vasculature

**FIGURE 6 ∣:**
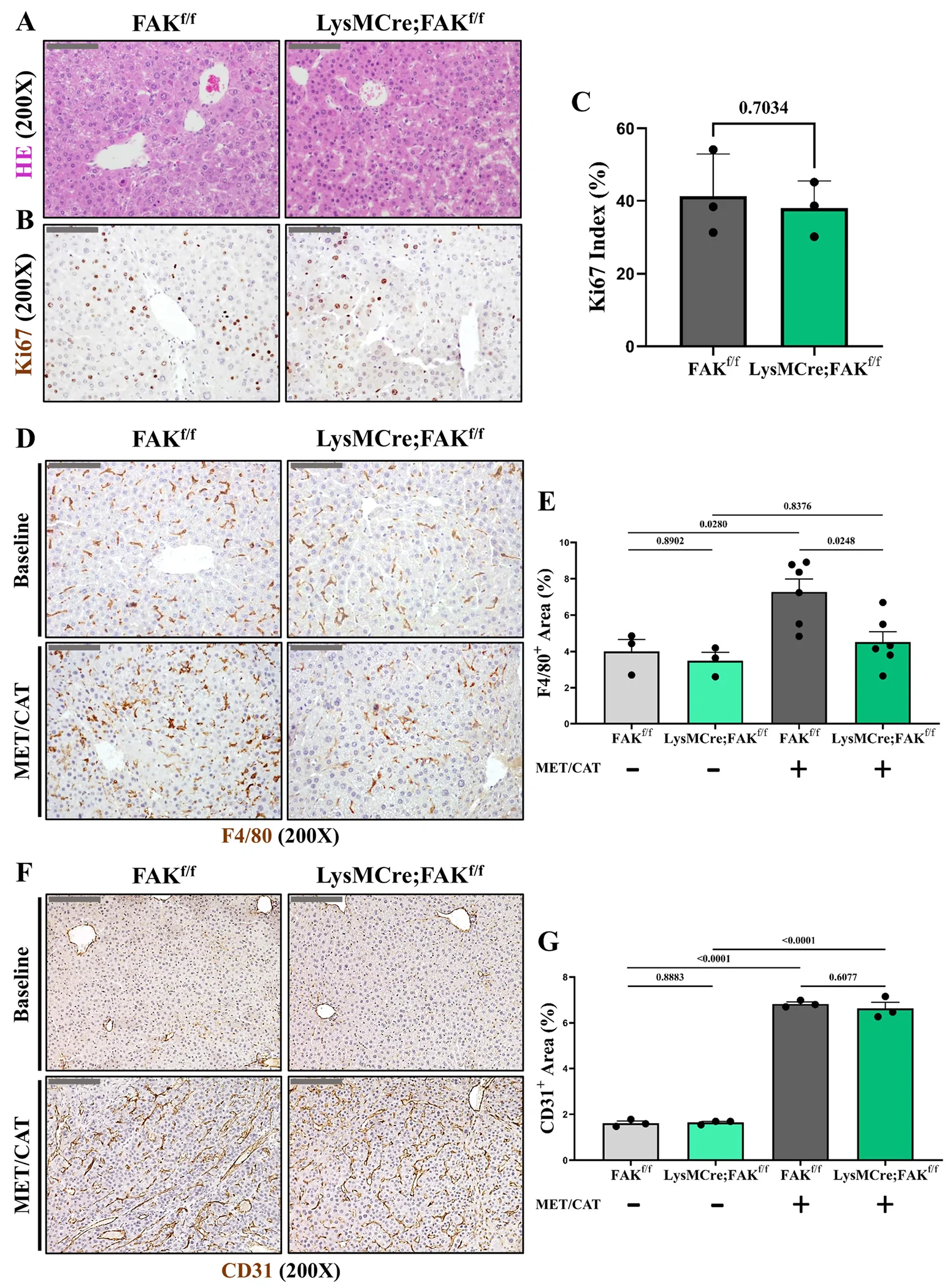
Myeloid FAK deletion reduces macrophage accumulation without affecting tumor proliferation. (A) Representative H&E-stained tumor sections from FAK^f/f^ and LysMCre;FAK^f/f^ mice. (B) Representative Ki67 IHC images of tumor sections. (C) Quantification of Ki67 index (%), *n* = 3 per genotype. Unpaired two-tailed *t*-test used; *p* = 0.7034. (D) Representative F4/80 IHC images from liver sections under baseline (*n* = 3) and tumor conditions (*n* = 6) following MET/CAT HTVi. (E) Quantification of F4/80 positive area (%) across groups. (F) Representative CD31 IHC images from liver sections under the same conditions as in (D). (G) Quantification of CD31-positive area (%) across groups. Data are presented as mean ± SEM, with each data point representing one individual sample. Scale bars = 100 μm. IHC quantification was performed at 10 random nonoverlapping 200× fields per sample, analyzed via ImageJ. F4/80 and CD31 quantification was analyzed via two-way ANOVA with Holm-šídák-corrected post hoc tests; *p* values are reported in the graph. [Color figure can be viewed at wileyonlinelibrary.com]

Given the absence of an overt survival or tumor burden phenotype, we next asked whether myeloid FAK deletion altered more granular features of the tumors and TME. Microscopic examination of H&E-stained sections revealed a similar tumor architecture across genotypes. Histologically, the livers contained numerous enlarged nodular regions of hepatocyte proliferation composed of highly atypical hepatocytes with enlarged, pleomorphic, hyperchromatic nuclei, nested and tra-becular growth patterns, prominent nucleoli, and frequent mitoses. These histological findings were consistent with a moderately differentiated HCC (Figure 6A). Ki67 indices were comparable between genotypes (*p* = 0.7034; Figure 6B,C). In contrast, F4/80^+^ macrophage accumulation differed between genotypes in a tumor-dependent manner. Baseline F4/80^+^ area was similar between genotypes. Following MET/CAT tumor induction, tumor tissue from FAK^f/f^ control mice showed significantly increased F4/80^+^ area compared with baseline liver tissue (*p* = 0.0280; Figure 6D,E). FAK deletion in myeloid cells attenuated this tumor-associated increase (*p* = 0.0248; Figure 6D,E).

Angiogenesis was enriched among FAK-correlated myeloid programs (Figure 3D), and *VCAN*^+^ macrophages were predicted to signal to endothelial cells via VEGF (Figure 3E). Given that myeloid FAK deletion reduced macrophage accumulation in LysMCre;FAK^f/f^ tumors, we performed IHC staining for the endothelial cell marker CD31 to test whether tumor vascularization was affected. CD31^+^ vascular area was significantly increased in tumor tissue compared to baseline liver in both FAK^f/f^ and LysMCre;FAK^f/f^ mice (*p* < 0.0001), consistent with prior reports [47]. However, the CD31^+^ vascular area was not significantly different between the genotypes at baseline (*p* = 0.8883) or in the tumor-bearing state (*p* = 0.6077; Figure 6F,G). Together, myeloid FAK deletion reduced macrophage accumulation in MET/CAT-driven HCC but did not translate into changes in tumor burden, proliferation, or vascular area.

## 4 ∣ Discussion

In this study, we investigated whether myeloid FAK contributes to HCC progression. Human transcriptomic analyses placed *PTK2* within a clinically relevant myeloid-rich context and identified a *PTK2*-enriched macrophage state marked by hypoxia and inflammatory transcriptional signatures. Functional studies using LysMCre;FAK^f/f^ mice showed that myeloid FAK deletion reduced macrophage accumulation, but did not alter survival, tumor burden, or proliferation in MET/CAT-driven HCC.

Focusing first on human data, the single-cell analyses indicate that *PTK2* expression in HCC extends beyond tumor cells and into specific myeloid populations. Among the refined subclusters, *VCAN^+^* macrophages showed the strongest *PTK2*-associated transcriptional signals enriched for hypoxia, glycolysis, and inflammatory response. At the bulk transcriptomic level, TCGA-LIHC analysis showed that patients with both high *PTK2* expression and high myeloid infiltration had worse overall survival, consistent with *PTK2* expression and a myeloid-rich context jointly marking a poor prognosis subset of HCC. Because these human analyses remain correlative and cannot directly measure FAK activity or define the cellular source of the survival association, we questioned whether myeloid FAK functionally contributes to HCC progression.

Myeloid FAK deletion did not alter baseline liver morphology or proliferation, indicating that myeloid FAK is not required for steady-state liver homeostasis. After MET/CAT tumor induction, LysMCre;FAK^f/f^ mice developed tumors with comparable growth kinetics, liver-to-body weight ratios, and Ki67 indices. Despite the human association observed in the TCGA-LIHC analysis, myeloid FAK loss alone was insufficient to alter tumor growth, proliferation, or survival in this oncogene-driven model. This divergence likely reflects *PTK2* expression being a correlative, transcript-level proxy that may not predict functional dependence on myeloid FAK.

Among the examined features, reduced accumulation of F4/80^+^ macrophages within tumors was the most prominent. This aligns with established roles of FAK in macrophage adhesion, chemotaxis, and directional motility. Owen and colleagues showed that myeloid FAK deletion in primary bone marrow-derived macrophages impaired lamellipodial stability, adhesion turnover, and monocyte recruitment to inflammatory sites in vivo [22]. These findings are consistent with a role for FAK in the reduced macrophage accumulation we observed in LysM-Cre;FAK^f/f^ tumors, although the inflammatory context differs. The transcriptional signatures of the *VCAN*^+^ macrophage population reinforce this interpretation. Glucose-uptake and glycolytic genes (*SLC2A3, HK2, PDK1*) and hypoxia-response genes (*EGLN3, P4HA1, ERO1A*) were among the significantly upregulated genes, indicating a macrophage state adapted for energy production under hypoxic conditions. Glycolysis supplies the ATP that powers actin-driven macrophage migration [48]. The glycolytic reprogramming of this state and its FAK-dependent accumulation could help explain the observed migratory phenotype. In fact, the absence of a baseline liver phenotype suggests that myeloid FAK may be most relevant under conditions of tumor-associated inflammation and remodeling.

Because CellChat predicted *VCAN^+^* macrophages to communicate with endothelial cells via VEGF and angiogenesis was enriched in FAK-correlated myeloid programs, we assessed whether reduced macrophage accumulation altered the tumor vasculature. Myeloid FAK deletion did not alter CD31^+^ vascular area between genotypes at baseline or in MET/CAT tumors. This disconnect could be partly explained by prior work showing that perturbing a myeloid angiogenic program can uncouple the vessel measurements from changes in tumor growth [49]. Shifts in TAM metabolism can also remodel vessel morphology and function while leaving vessel density unchanged [50]. These findings suggest that losing a single macrophage population may not change CD31^+^ vascular area. These results, however, do not demonstrate direct effects on active vascular remodeling or angiogenic signaling, as CD31 marks endothelial cells rather than vessel maturity, perfusion, or angiogenic activity.

Several mechanisms could explain the outcomes. The MET/CAT model is aggressive and strongly oncogene-driven, potentially limiting the impact of perturbing a single myeloid signaling axis. β-catenin activation has also been directly linked to immune exclusion in HCC, with impaired dendritic cell recruitment and resistance to anti-PD-1 therapy [51], which may diminish reliance on macrophage-mediated tumor support relative to other molecular subtypes. Compensation through parallel pathways is also plausible. Tumors can receive pro-angiogenic signals from other stromal cell types in the TME [52]. Additionally, reduced macrophage infiltration can be functionally compensated where MMP-9^+^ neutrophils can sustain angiogenesis when macrophage recruitment is impaired in a cervical cancer model [53]. Combined FAK and PYK2 loss does not produce a greater impairment in macrophage invasion than deletion of either kinase alone, suggesting these kinases operate within overlapping motility pathways [22]. Src-family kinases, integrin-associated signaling, and CSF1R-mediated programs may additionally sustain macrophage function in the absence of FAK [54]. Finally, FAK activity in nonmyeloid compartments, such as tumor cells, endothelial cells, and fibroblasts, also remains intact and may independently support tumor growth. Notably, endothelial cells and hepatic stellate cells showed comparable *PTK2* expression to tumor cells in the PRHCCdb data set (Figure 1A), suggesting these compartments may represent additional FAK-associated tumor-promoting niches worth exploring in future studies. Consistent with this, prior work from our group showed that hepatocyte-specific FAK deletion suppresses MET/CAT-driven HCC and prolongs survival in this same model [17, 18], reinforcing tumor cell-intrinsic FAK as the dominant driver of progression.

While our findings provide important insights, several limitations should be acknowledged. The single-cell analyses remain correlative. The *VCAN*^+^/*COLEC12*^+^ state was concentrated in a small number of tumors, so the donor-level pseudo-bulk validation relied on only 2 VCAN^+^ aggregates and 19 comparators. Although fold-changes were highly concordant with the cell-level analysis, this imbalance limits the power for individual genes and the generalizability for comparison, which requires further confirmation in additional cohorts. The FAK pathway module score reflects transcriptional enrichment rather than kinase activity, and CellChat predictions require experimental validation. The in vivo work was limited to a single HCC model. While typically used for macrophage studies, *LysM*-Cre targets multiple myeloid lineage cells without distinguishing infiltrating macrophages from resident Kupffer cells in the liver. The recombination efficiency with this Cre model also varies across different myeloid subsets [55]. Future studies incorporating approaches such as *Cx3cr1*-CreER or *Ccr2*-Cre, flow cytometry, or spatial transcriptomics may help resolve which myeloid populations are most affected by myeloid FAK deletion and whether their polarization, cytokine profiles, or cellular cross-talk networks are altered. Testing therapeutic combinations with FAK inhibitors or immune checkpoint blockade could also clarify whether myeloid FAK becomes more functionally relevant under treatment pressure or in less aggressively driven models.

## 5 ∣ Conclusion

Together, these findings identify myeloid FAK as a regulator of macrophage accumulation in HCC but demonstrate that its loss alone was insufficient to alter tumor burden, vasculature, proliferation, or survival in the MET/CAT HCC model. The human transcriptomic data link *PTK2*- enriched macrophages with hypoxia and inflammatory programs, signatures that often coincide with tumor-promoting features of the TME. Collectively, these results support a context-dependent role for myeloid FAK in shaping the tumor macrophage compartment and underscore the need to evaluate this axis within broader therapeutic and combinatorial frameworks.

## Figures and Tables

**TABLE 1 ∣:** Genotyping primers.

Primers	Sequence
*Lyz2* F1	5′-AAGGAGGGACTTGGAGGATG-3′
*Lyz2* R1 (WT)	5′-GTCACTCACTGCTCCCCTGT-3′
*Lyz2* R2 (Mut)	5′-ACCGGTAATGCAGGCAAAT-3′
*Ptk2* F1	5′-GAGAATCCAGCTTTGGCTGTTG-3′
*Ptk2* R1	5′-GAATGCTACAGGAACCAAATAAC-3′

Abbreviations: *Lyz2*, lysozyme 2; Mut, mutant; *Ptk2*, protein tyrosine kinase 2; WT, wild type.

**TABLE 2 ∣:** Key reagents, antibodies, and plasmids.

Reagents	Species	Catalog #	Source	Application
Mouse GM-CSF Recombinant Protein, PeproTech	Mouse	315-03	Thermo Fisher Scientific	Cell culture
RPMI-1640 (Cytiva HyClone)	N/A	SH30027FS	Cytiva	Cell culture
FAK antibody	Rabbit	3285	Cell Signaling	WB
GAPDH antibody	Mouse	60004-1-Ig	Proteintech	WB
MET antibody	Rabbit	8198	Cell Signaling	WB
β-Catenin antibody	Mouse	610153	BD Biosciences	WB
Goat-anti-Rabbit IgG (H + L), HRP	Goat	PI31462	Invitrogen	WB
Goat-anti-Mouse IgG (H + L), HRP	Goat	PI31432	Invitrogen	WB
ECL substrate (SuperSignal West Pico PLUS)	N/A	34578	Thermo Fisher Scientific	WB
F4/80 antibody	Rat	MCA497GA	Bio-Rad	IHC
Ki67 antibody	Rabbit	12202	Cell Signaling	IHC
CD31 antibody	Rabbit	77699	Cell Signaling	IHC
Goat-anti-Rabbit IgG (H + L), Biotin	Goat	31822	Invitrogen	IHC
Goat-anti-Rat IgG (H + L), Biotin	Goat	31830	Invitrogen	IHC
DAB Substrate Kit, Peroxidase (HRP)	N/A	SK-4100	Vector Laboratories	IHC
ABC-HRP Kit, Peroxidase	N/A	PK-6100	Vector Laboratories	IHC
Permount Mounting Medium	N/A	SP15-500	Fisher Scientific	IHC
pSB-EF1α > hMET	Human	VB231106-1311wdq	VectorBuilder	HTVi
pSB-EF1α > ΔN90-β-catenin	Human	VB231031-1458txy	VectorBuilder	HTVi
HSB2	N/A	N/A	Generated in-house	HTVi

Abbreviations: ABC-HRP, avidin-biotin complex horseradish peroxidase; CD31, endothelial marker; DAB, 3,3′-diaminobenzidine; ECL, enhanced chemiluminescence; EF1α, elongation factor 1 alpha; FAK, focal adhesion kinase; F4/80, macrophage marker; GAPDH, glyceraldehyde 3-phosphate dehydrogenase; GM-CSF, granulocyte-macrophage colony-stimulating factor; H + L, heavy and light chains; HSB2, hyperactive Sleeping Beauty transposase; HTVi, hydrodynamic tail vein injection; IgG, immunoglobulin G; IHC, immunohistochemistry; Ki67, proliferation marker; MET, MET proto-oncogene receptor tyrosine kinase; N/A, not applicable; pSB, Sleeping Beauty transposon plasmid; RPMI, Roswell Park Memorial Institute; WB, western blot.

**TABLE 3 ∣:** Gene modules and pathway collection for scRNA-seq analysis.

Module	Genes included	Usage
Canonical myeloid subtype markers	*FOLR2, TIMD4, S100A8, S100A9, FCN1, VCAN, AQP9, CD300E, CXCL8, IL1B, TREM1, CD1C, FCER1A, CLEC10A, MKI67, TOP2A, IFIT1, IFI44L, CHGA, ALB, APOC3*	Myeloid subtype validation and contamination removal
Myeloid lineage	*LYZ, AIF1, CD68, CSF1R, TYROBP, C1QA, C1QB, C1QC, FCGR3A*	Myeloid subtype annotation
Cycling/proliferating myeloid markers	*MKI67, TOP2A*	Lineage validation
FAK pathway score	*PTK2, PXN, VCL, TLN1, ILK, BCAR1, CRK, CRKL, PIK3R1, PIK3CA, AKT1, MTOR, RPS6KB1, SRC, PTPN11, GRB2, SOS1, HRAS, RAF1, MAP2K1, MAPK3, RAC1, CDC42, PAK1, RHOA, ROCK1, MYH9, ACTN1, FN1, ITGB1, ITGAV*	Calculate FAK pathway module score for myeloid subtypes
GSEA	MSigDB Hallmark, KEGG Medicus, Reactome	Pathway enrichment analysis

*Note:* Gene names are listed as official gene symbols.

Abbreviations: FAK, focal adhesion kinase; GSEA, gene set enrichment analysis; KEGG, Kyoto Encyclopedia of Genes and Genomes; MSigDB, Molecular Signatures Database.

## Data Availability

Computational scripts and processed differential expression, pathway enrichment, CellChat, TCGA-LIHC output tables are available on Zenodo at DOI: 10.5281/zenodo.20090063. PRHCCdb was accessed at (https://db.cngb.org/PRHCCdb/), public scRNA-seq data were downloaded from GSE151530, and TCGA-LIHC data were obtained from the Genomic Data Commons.

## Abbreviations:

BH: Benjamini–Hochberg
CAT: constitutively active form of β-catenin (ΔN90-β-catenin)
CD31: cluster of differentiation 31
DEG: differentially expressed gene
ECM: extracellular matrix
FAK: focal adhesion kinase
FAK^f/f^: FAK^flox/flox^
FDR: false discovery rate
GM-CSF: granulocyte-macrophage colony-stimulating factor
GSEA: gene set enrichment analysis
HCC: hepatocellular carcinoma
HTVi: hydrodynamic tail vein injection
H&E: hematoxylin and eosin
IHC: immunohistochemistry
LIHC: liver hepatocellular carcinoma
NES: normalized enrichment score
NF-κB: nuclear factor kappa-light-chain-enhancer of activated B cells
PTK2: protein tyrosine kinase 2
scRNA-seq: single-cell RNA sequencing
TAM: tumor-associated macrophages
TCGA: The Cancer Genome Atlas
TME: tumor microenvironment
UMAP: uniform manifold approximation and projection
VEGF: vascular endothelial growth factor
